# Applications of Artificial Intelligence, Machine Learning, Big Data and the Internet of Things to the COVID-19 Pandemic: A Scientometric Review Using Text Mining

**DOI:** 10.3390/ijerph18168578

**Published:** 2021-08-13

**Authors:** Ignacio Rodríguez-Rodríguez, José-Víctor Rodríguez, Niloofar Shirvanizadeh, Andrés Ortiz, Domingo-Javier Pardo-Quiles

**Affiliations:** 1 Protein Structure and Bioinformatics Resech Group, Department of Experimental Medical Science, Lund University, SE-221 84 Lund, Sweden; niloofar.shirvanizadeh@med.lu.se; 2 Departamento de Tecnologías de la Información y las Comunicaciones, School of Telecommunications Engineering, Universidad Politécnica de Cartagena, 30202 Cartagena, Spain; domingo.pardo@upct.es; 3 Departamento de Ingeniería de Comunicaciones, School of Telecommunications Engineering, Universidad de Málaga, 29071 Málaga, Spain; aortiz@ic.uma.es

**Keywords:** COVID-19, artificial intelligence, machine learning, scientometrics, text mining, VOSviewer

## Abstract

The COVID-19 pandemic has wreaked havoc in every country in the world, with serious health-related, economic, and social consequences. Since its outbreak in March 2020, many researchers from different fields have joined forces to provide a wide range of solutions, and the support for this work from artificial intelligence (AI) and other emerging concepts linked to intelligent data analysis has been decisive. The enormous amount of research and the high number of publications during this period makes it difficult to obtain an overall view of the different applications of AI to the management of COVID-19 and an understanding of how research in this field has been evolving. Therefore, in this paper, we carry out a scientometric analysis of this area supported by text mining, including a review of 18,955 publications related to AI and COVID-19 from the Scopus database from March 2020 to June 2021 inclusive. For this purpose, we used VOSviewer software, which was developed by researchers at Leiden University in the Netherlands. This allowed us to examine the exponential growth in research on this issue and its distribution by country, and to highlight the clear hegemony of the United States (USA) and China in this respect. We used an automatic process to extract topics of research interest and observed that the most important current lines of research focused on patient-based solutions. We also identified the most relevant journals in terms of the COVID-19 pandemic, demonstrated the growing value of open-access publication, and highlighted the most influential authors by means of an analysis of citations and co-citations. This study provides an overview of the current status of research on the application of AI to the pandemic.

## 1. Introduction

In March 2020, the World Health Organization (WHO) declared COVID-19, the disease caused by the SARS-CoV-2 virus, to be a pandemic, which has since claimed the lives of millions of people worldwide.

Since then, researchers around the world have been working to understand how the virus functions in an attempt to stop its spread. In this regard, the contribution of strategies supported by artificial intelligence (AI) and other emerging technologies has been unquestionable. Intelligent data analysis, which has become possible due to the development of high-performance computing resources (cloud computing) and recent improvements in deep learning algorithms, machine learning and neural networks, allows researchers to successfully process large amounts of data and to extract knowledge. AI can contribute to these objectives by providing efficiency and speed in terms of obtaining results, as well as by generating new solutions and new lines of research.

Since the beginning of the pandemic, scientific production in many different areas, sometimes supported by AI, has continued to grow, and researchers have begun to address issues related to detection and transmission, vaccines, treatments, and, more generally, appropriate management of this exceptional situation. This has led to explosive growth in the scientific literature, making it very difficult to understand how research is developing, particularly with respect to the areas in which research is being carried out, which are the hot topics, and which authors or countries are leading this work.

A huge number of technological methodologies are arising to manage the effects of the COVID-19 pandemic. Among them, emerging technologies including Internet of Things (IoT), AI, blockchain, and cutting-edge media transmission networks such as 5G have been at the bleeding edge [1].

The Internet of Medical Things (IoMT), additionally alluded to as the medical care IoT, is a blend of clinical gadgets and programming applications offering broad medical care services that are associated with the medical services IT frameworks. Due to their capacity to gather, investigate, and send wellbeing information proficiently, the medical services area has understood the extraordinary capability of IoMT innovations. The act of utilizing IoMT advancements to work with distant patient observing is called telemedicine. It permits clinicians to assess, analyze, and treat patients without requiring any actual association with them [2]. Following the episode of the exceptionally infectious COVID-19, some IoMT tech and telemedicine platforms have been studied as a solution [3].

Likewise, wearables can be a legitimate medical technology, because of the biosensors that are installed inside. The capacity to screen individuals’ actual wellbeing, alongside their feelings of anxiety, has made wearables an optimal innovation for reception in the medical services area [4].

Additionally, we can name blockchain, as it is acquiring more importance every day on account of its wide applications in different backgrounds [5]. Seeing its utility, various researchers across the globe have begun utilizing blockchain to assemble applications that can help in countering the COVID-19. These applications plan to resolve a vital issue, which is the lack of integration of verified data sources such as vaccination certificates [6]. Blockchain can approve consistently evolving information. This component can end up being very important in dealing with the quickly raising COVID-19 circumstance [7].

Concerning following the applications, advancements such as Bluetooth and other short-distance communications can be useful [8]. It is quite possibly the most interesting innovation utilized for exact vicinity estimation. Additionally, it is one of the most unintrusive innovations, as it does not screen the specific area of a cell client but instead the relative distance between a gadget and that of another [9].

AI and Machine Learning (ML), whenever utilized appropriately, are compelling technologies against the COVID-19 pandemic [10]. They can be utilized for different purposes, such as infection reconnaissance, hazard forecast, clinical analysis, infection demonstrating or implementing the public approaches measures.

In another vein, 5G refers to the fifth generation of wireless communication technology regarding mobile networks globally [11]. Along with other corresponding innovations such as IoT and AI, 5G organization innovation can possibly alter the medical care area in the COVID-19 pandemic by giving better help to the forefront staff and by providing further developed infection following, patient checking, information assortment, and investigation [12].

The scientific studies available in this area are scattered, and their numbers are overwhelming, making it hard for researchers to obtain a structured view of the state of the art. Fortunately, interesting reviews quickly took over this situation, providing a complete overview, updated to the first months of the pandemic, and even creating an interesting repository of papers on data science-based applications that can help in the COVID-19 pandemic [13].

Scientometric analysis is a technique that can provide a macroscopic view of a large amount of academic literature; through a quantitative analysis using text mining techniques, it is possible to map the scientific development of a given field of research. In this way, it is possible to identify patterns related to authors, journals, countries, and the issues on which research is focused and which have already been surpassed.

Regarding the present topic, scientometric analysis can provide an overview of those areas that are multidisciplinary or are experiencing greater inter-collaboration in terms of the management of the current pandemic, and which are supported by emerging technologies. Automated techniques for the analysis of scientific literature can be used to identify research directions and the current gaps in a given field, and can consequently help in the decision-making process related to COVID-19 and support funding agencies in assigning funding.

These techniques have already been used throughout the months of pandemic evolution with great success. Several studies have used scientometrics to condense and define trends in COVID-19 related research. One of the best works in this regard is presented by Colavizza et al. [14], using Web of Science as a data source, updated to 2020, and including research related to several coronaviruses. Duan and Qifan [15] have also conducted a study on scientific collaboration on COVID-19, covering the first six months of 2020. They also use WoS as a data source. Haghani and Varamini [16] also use WoS and go forward to the end of August 2020 to study scientific developments related to the pandemic using scientometrics. Hossain [17] also reviewed 422 papers up to April 2020 using the same source of data. On the other hand, Pal [18] used Scopus as a database instead, with searches up to May 2020.

The above studies, being of exceptional quality, cover COVID-19 research in a general way, not focusing on a specific field of research. To the authors’ best knowledge, there has so far been no study that focuses on the applications of the so-called emerging technologies, headed by artificial intelligence, studying the different topics to which it is applied and the characteristics of this particular scientific production. On the other hand, the pace at which research on the pandemic is advancing requires constant updating, so this work aims to update previous work up to the end of June 2021. Thus, the rationale of this article is twofold: on the one hand to provide an in-depth analysis of how machine learning, big data analysis and other related disciplines are helping in the pandemic, and at the same time, on the other hand, to present it up to date to the present time.

Section 2 introduces the theoretical fundamentals of scientometrics and the methodological approach, and especially the data collection and treatment processes. In Section 3, the main results are presented with a discussion. Section 4 contains our conclusions and suggests future lines of interest.

## 2. Materials and Methods

### 2.1. Scientometrics and Text Mining

Scientometrics is the study of the quantitative aspects of the production, spread and use of scientific information, with the aim of achieving a better understanding of the mechanisms of scientific research and its evolution [19]. It therefore represents a research technique in the information and library sciences that can be used to examine bibliographic data, such as authors, year of publication, country of origin and others, through the use of quantitative tools such as text mining [20]. This type of analysis is very advantageous in terms of inferring a representative outline of a set of scientific documents. According to the literature, many scientometrics tools have been used to analyze a huge variety of aspects, including topics and keywords [21], institutions, authors and countries. There are many different indicators that can be used to measure the importance of this information, for example the number of papers and citations [22]. This work applies several of these indicators in order to offer a diverse range of views, so that the reader can comprehend the outcomes in terms of their particular advantages and needs.

There is no general agreement on the best way to correctly evaluate a set of scientific documents. From an overall perspective, the two main metrics used to estimate research output are productivity and influence [23]. Productivity is frequently measured based on the total amount of publications, while influence is reflected by the quantity of citations. Nonetheless, there are different indicators for doing as such and numerous extraordinary circumstances may emerge.

### 2.2. Software

We carried out scientometric mapping and text mining using VOSviewer software, which was developed at Leiden University in the Netherlands [24]. VOSviewer uses text mining to identify publication keywords and then uses a mapping technique called visualization of similarities (VOS) to draw bibliometric maps called landscapes [25]. Landscapes can be displayed in various ways to allow the researcher to infer the different characteristics of the content of the research papers.

VOSviewer compiles bibliographic data and offers graphical maps that represent bibliographic coupling [26], co-citations [27], co-authorship, and co-occurrence of author keywords.

The software uses the Leiden algorithm to find well-connected clusters in networks. This algorithm outperforms the Louvain algorithm by solving some of its shortcomings: the clusters it finds can be arbitrarily connected. The Leiden algorithm yields communities that are ensured to be connected. What is more, when the Leiden algorithm is used iteratively, it converges to a partition in which all subsets of all populations are locally optimally assigned [28].

VOSviewer uses for the graph layout the visualization of similarities (VOS) algorithm, introduced in 2007 by the authors Van Eck and Waltman [29]. VOS provides a low-dimensional visualization in which objects are located in such a way that the distance between any pair of objects reflects their similarity as accurately as possible. The idea of VOS is to minimize a weighted sum of the squared Euclidean distances between all pairs of objects. The higher the similarity between two objects, the higher the weight of their squared distance in the summation.

### 2.3. Indicators

In order to provide an adequate summary of the provenance of the research related to intelligent data analysis and COVID-19, the following indicators were studied.

#### 2.3.1. Production and Chronology

The first piece of information to be analyzed was the amount of scientific production related to the applications of AI to COVID-19 management. The most productive countries could be identified in this way and could be analyzed in terms of their gross domestic product (GDP). The distribution of this research over time can give us an idea of the extent to which it is a hot topic in the scientific field.

#### 2.3.2. Topics Analysis

This indicator is used to extract the hidden topics addressed in the bibliographic materials of interest using a topic mapping technique. This examination applies factual methodology to turn idle (unobvious or undetectable) topics within enormous bodies of bibliographic materials into unequivocal visual displays of clusters of subjects and the associations between them. Topic mapping analysis is a promising tool that is used in scientometrics and text mining [30]. This technique exploits the dissimilarities between probability distributions, or in other words, the distribution of a given semantic element over the group of all topics, and the distribution of all semantic elements over the group of all topics [31]. When these distributions are very divergent, we can conclude that a semantic element is likely to characterize a certain idea; on the other hand, if the distributions are closely related, this means that a semantic element does not represent a specific concept. The relationships among keywords are calculated based on the number of times they co-occur throughout the articles: a larger number of papers in which two terms co-occur indicates a more robust relationship between the two terms. Based on the results, the terms can be grouped into clusters to form a map, using a technique called VOS [32].

#### 2.3.3. Citation and High Cited Elements

The most frequently cited journals, papers and authors can be used to identify the most important elements of research in the field of COVID-19 and associated disruptive technologies.

#### 2.3.4. Co-Citation Analysis

A co-citation analysis applies weights that correspond to the strength of co-citations, with a larger value indicating a greater tendency for authors or journals to be cited together in the same article. The idea behind co-citation analysis is that articles by researchers who are often co-referenced are likely to address similar or related ideas [33]. The Java-based VOSviewer software creates a co-citation matrix using Van Eck’s [34] clustering technique to display clusters of closely related publications. Co-citation analysis is conducted based on a minimum of 20 citations. The purpose of this threshold is to reduce the amount of disorder in the data visualization. In this sense, some other lower citation thresholds (e.g., at five, 10 or 15 citations) were also fixed in this work to obtain an optimum. This co-citation process can be performed at either the author or journal level; in the former, a co-citation value is computed based on the relationships between articles by a given author, and in the latter, based on the relationships between journals. The resulting co-citation structures can provide rich insight into the field of applications of AI to COVID-19.

#### 2.3.5. Overlay Visualization

This type of analysis makes it possible to superimpose other types of information, such as the year of publication, on either of the two previous analyses described above, meaning that the trajectory of a given line of research or collaborations can be seen. This strategy is one of the most important tools that can be used in scientometrics [35] for automatic trend detection. Using this approach, it is possible to see at a glance the evolution of a subject. VOSviewer can be used to plot a map showing the relationships between certain elements and then to overlay other data points with added information (e.g., citation impact, age of publication, etc.).

### 2.4. Data Acquisition

#### 2.4.1. Sources of Data

Web of Science (WoS) and Scopus are the two generally recognized bibliographic databases as the most comprehensive data sources for various purposes [36]. WoS was the first comprehensive international bibliographic database. Thus, over time, it has become the most influential bibliographic data source traditionally used for bibliometric analysis [37]. Over the years Scopus has carved out its place as a comprehensive bibliographic data source and has proven to be reliable and in some cases even better than WoS [38].

WoS is a multidisciplinary and selective database made up of a large number of specialized indices. The main part of the WoS platform is the Core Collection (WoS CC), which comprises six main citation indices: Science Citation Index Expanded (SCIE); Social Science Citation Index (SSCI); Arts and Humanities Citation Index (AandHCI); Conference Proceedings’ Citation Index (CPCI); Book Citation Index (BKCI); and the recently established Emerging Sources Citation Index (ESCI) [39].

Scopus is a similar multidisciplinary database [40]. Scopus also contains content from many specialized databases such as Embase, Compendex, World Textile Index, Fluidex, Geobase, Biobase and Medline [41], the content of which is integrated and similarly available.

There are also some other databases such as Google Scholar (GS) [42]. The main advantage of this DB is that no subscription is required, and all content is freely available to all users. GS also offers much broader and deeper general content, although not clearly defined. The free access and full coverage give GS a great advantage over WoS and Scopus. This also makes GS less reliable as a source of bibliographic data. The main disadvantages of GS are the lack of transparency, stability and precision [43]. Therefore, GS is not discussed in this article. There are other sources of data that can be more beneficial for certain purposes. Many of these are also relatively new and free products such as Microsoft Academic, CrossRef, ResearchGate, OpenCitations, etc. [44]. Therefore, their validity is still questionable.

Scopus offers broader overall coverage compared to WoS CC and has been confirmed multiple times, both through previous and latest comparisons of content coverage. In general, the contents indexed in WoS and Scopus also showed a lot of overlap, with Scopus indexing a larger number of unique sources that were not recorded by WoS [45]. A large-scale comparison at the journal level has shown that WoS and Scopus gravitate towards the natural sciences, engineering, and biomedical research, and Scopus offers broader coverage of all areas studied, particularly biomedical research [46]. However, the absolute majority of these studies report better coverage of Scopus from all important disciplines compared to WoS [47].

For all the above reasons, the Scopus database was used to search for specific applications of intelligent data analysis to the management of COVID-19. However, in the following section we will make a comparison of all the data obtained both from this database and WoS, as well as specific comparisons of results in order to verify the conclusions that will subsequently be drawn.

#### 2.4.2. Collected Data

The data were collected from Scopus by 30 July 2021, and all available publications were considered, up to 18,955 manuscripts. Since the outbreak of SARS-CoV-2 virus occurred at the beginning of the previous year, the results covered the year 2020 and the first six months of 2021.

Numerous fields were exported for each record, including the authors, the country of origin, the title, abstract, keywords, date of publication and the journal. Table 1 summarizes the searches that were performed and the search terms used.

To identify all the records of interest, searches were made that connected COVID-19 with AI-related concepts. The most general concepts relating to artificial intelligence have been sought, as reviewed in recent literature [48] and completed with specific reviews about emerging technologies [49,50].

In addition to more general areas of application, we included several searches for specific algorithms and applications [51], although some of these gave only marginal results. Other search terms that returned a large number of scientific papers (such as ‘clustering’ or ‘dataset’) were excluded, as in some cases the appearance of this word did not necessarily mean that the paper was related to the topic of interest. Other much more unusual and specific terms report very few results, and, on the other hand, such results already appeared in other more general searches, such as “machine learning”, “artificial intelligence” or “big data”.

Papers appearing in several sub-searches were counted only once.

The data were exported into text format, both in total and broken down by month, and were prepared for import into VOSviewer after filtering outliers and some incomplete records.

The same search has been carried out with the same terms in WoS. All of them have also been exported so that we can later compare them with the Scopus results.

## 3. Results and Discussion

### 3.1. Production and Chronology Analysis

**Figure 1:**
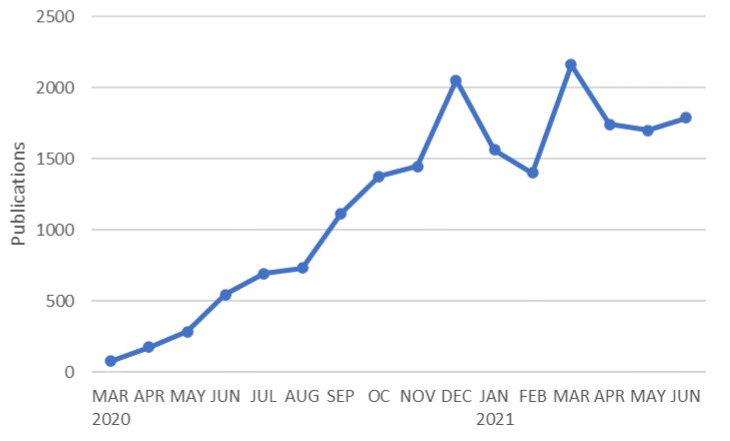
Temporal evolution of the number of publications related to emerging technologies and COVID-19 (source Scopus).

As the pandemic has progressed and the body of research related to COVID-19 has consequently increased, the use of intelligent data analysis has intensified. Of the total number of manuscripts identified in Scopus at the data acquisition stage (18,955), it can be seen that 8597 were generated during 2020, while in 2021 a total of 10,358 had already been published by the end of June. The introduction of vaccines and their proven efficacy, which has resulted in a decrease in the number of cases and better prognoses, has led to a normalization of the situation in many countries, and this seems to have led to a reduction in the number of manuscripts published. From Figure 1, we can see a slowdown in the number of papers after the initial explosion. It is important to bear in mind that 12,270 of the total number of papers were published in open access journals, according to the results produced by Scopus.

It should be noted that using the same search criteria, both terminological and temporal, in WoS the collected documents amount to 5938, of which 2576 were published in 2020 and 3362 correspond to the year 2021. This result confirms the observations made in the previous section, which indicated some observations on the greater coverage of Scopus compared to WoS. Of the total amount, 4847 correspond to articles in Open Access format.

As it is presumed to be more complete, the present study will be carried out using the data obtained from Scopus, with occasional comparisons with the WoS data.

**Figure 2:**
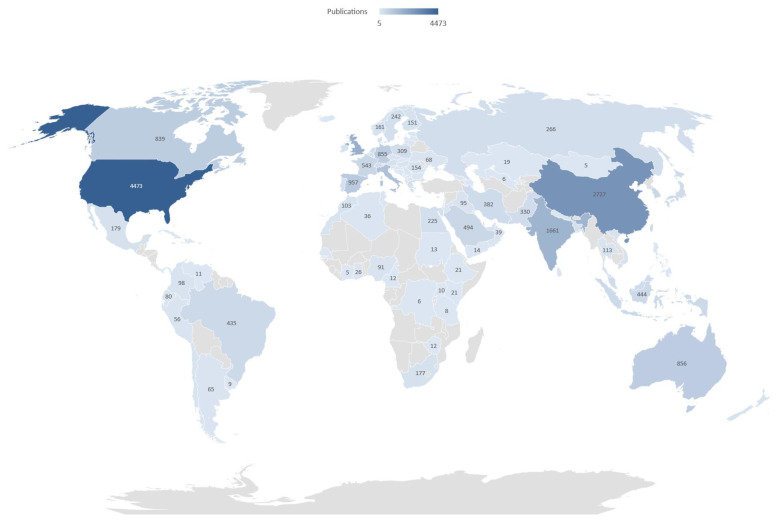
Number of COVID-19 and emerging technologies publications by country (source Scopus).

From Figure 2, it can also be seen that scientific production was clearly concentrated in the United States of America (4473) and China (2727). The United Kingdom has published 1843 papers and India 1661 scientific papers, followed by Italy. Other European countries have also generated a high level of scientific output related to the application of intelligent data analysis to COVID-19. The same ranking performed with WoS offers the same order for the first five positions.

**Figure 3:**
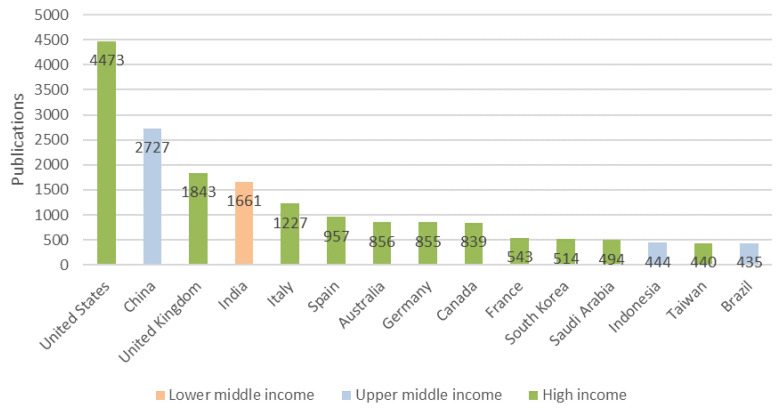
Top 15 most productive countries and their classification according to incomes (source Scopus).

It should be noted that the two countries with the highest scientific production also correspond to the two largest economies in the world, based on their GDP (USD 21,433,226,000,000$ and USD 14,342,903,006,431$, respectively, for the USA and China [52]). The 10 countries with the highest numbers of publications are all classified as high-income nations by the World Bank [53] with the exceptions of China and India, which are in the upper middle and lower middle income bands, respectively (Figure 3). In this ranking based on numbers of papers, the highest country that is classified as low income is Ethiopia, in 60th place, having published only 10 papers.

The distribution shown in Figure 3 broadly follows the pattern of global scientific publication (taking into account all disciplines and all subjects). Scopus provides with the country rank through its SCImago index. SCImago rank is one among the several types of quality measures Scopus provides. SCImago presents ranks making use of data supplied by Scopus.

**Figure 4:**
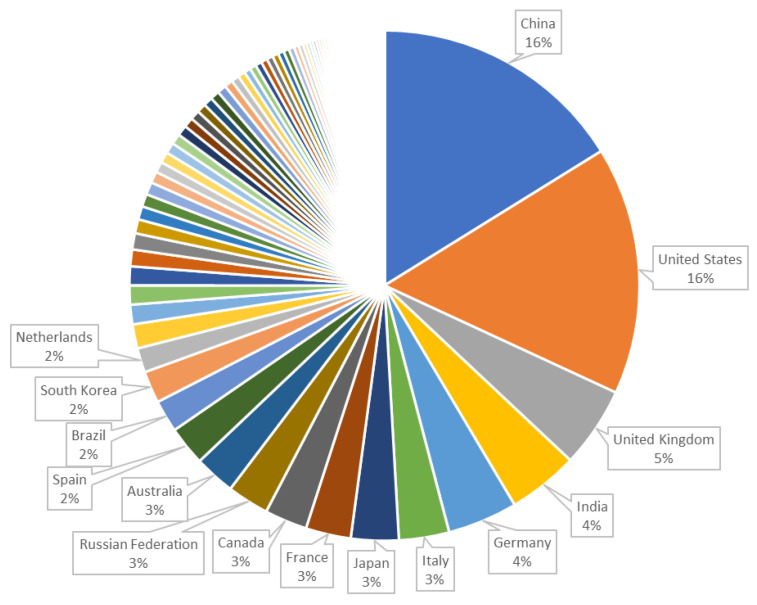
Top most productive countries in all scientific fields in 2020 (source Scopus).

Figure 4 shows the data for the year 2020 in order to make a comparison with the data obtained according to the above search criteria (Table 1).

It can be seen that the top positions are held by China and the USA, although when it comes to COVID-19 and emerging technologies, the USA leads very clearly, probably due to increased private investment. Following the data in Figure 4, in third and fourth place we find United Kingdom and India, as in Figure 3. From here on, the number of papers (in both cases) is very similar between the countries from fifth to fifteenth, and we see that Germany, Canada, France, South Korea, appear in both rankings. It should be noted that, in terms of COVID research, Italy and Spain have higher positions than in the general ranking, and we should not forget that the pandemic had a greater impact in Europe in these two countries.

In any case, we can conclude that the presence of a good previous research structure (in all fields and disciplines) has given support to the research that has been necessary to carry out quickly and urgently in an emergency situation such as this.

**Figure 5:**
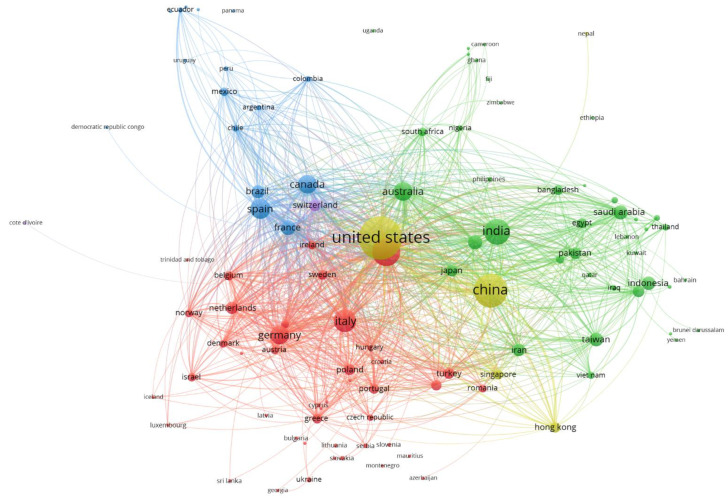
Co-authorships by countries (source Scopus).

After examining the amounts of scientific production, we investigated the main collaborations among countries (Figure 5; please note that the colors of the clusters are not related to the colors used in the previous figures).

Clusters were created based on the frequency of co-occurring terms indicating each country; the more often the words tend to co-occur they get painted into clusters. The size of each sphere represents the number of papers published by a country, while the thickness of each line illustrates the magnitude of the collaboration. It can be observed at a glance that these are dominated by two main scientific producers (yellow clusters) with a high level of collaboration between them, almost exclusively, with the collaboration of some leading places in technology and intelligent analysis, such as Hong Kong.

Other collaborative clusters seem to be organized mainly based on geographical area or cultural/social affinity. The red cluster comprises mainly Central European and Northern European countries, whereas the blue cluster relates to countries in the with Latin American authors. The green cluster includes Asian and some North African countries, as well as collaborations among Arab countries. We can therefore infer that collaborations are highly conditioned by geographical proximity, cultural issues and linguistic similarity.

**Figure 6:**
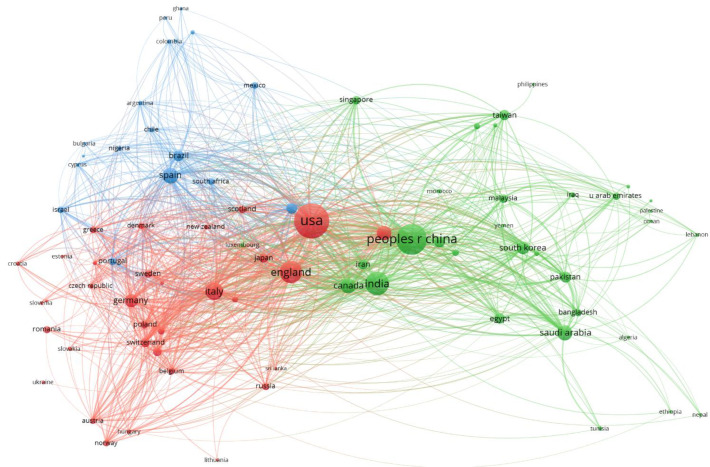
Co-authorships by countries (source WoS).

A very similar graph can be obtained with WoS data. The same collaborative clusters between countries and their interconnections can be observed, which is reflected in Figure 6. Again, the predominance of the United States and China is clear, as well as the different collaborations by country. Note the predominant role of Spain as a nexus between Europe and Latin America.

Obtaining similar results when using Scopus or WoS will verify that both samples, although very different in size, equally reflect equivalent information.

### 3.2. Topic Analysis

**Figure 7:**
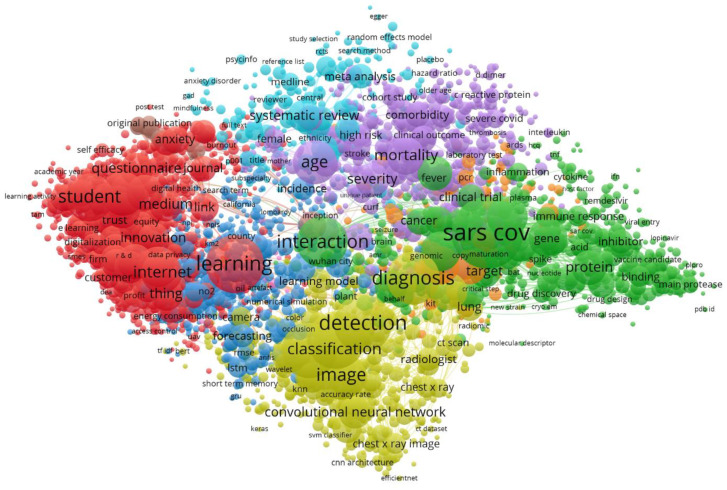
Topic mapping and clustering based on affinities (source Scopus).

A topic analysis was carried out with VOSviewer [54] to build a map representing the main relevant subjects and their relationships, using semantic analysis. In our case, the text mining algorithms identified 4485 relevant subjects using Scopus, and the clustering process classified these into six main clusters according to their similarities (Figure 7). Clusters were created based on the frequency of co-occurring terms: the more often the words tend to co-occur they get painted into clusters. The size of each circle represents the number of times that a word occurs. This automatic procedure was supported by the expertise of the authors in this field, which made it possible to identify the issues and gain a better understanding of the concept maps. As the present paper aims to provide an overview of almost 19,000 papers (Scopus), the following points are not intended to be an exhaustive review of all of the possible contributions, but rather to provide clarifying examples of the topics represented by each of the clusters.

The six clusters can be described as follows. Two main groups can be distinguished: the first three clusters (red, light blue, dark blue) represent topics related to AI applications and data analysis at large scales and at the social level, such as spread monitoring, the localization of outbreaks, social adaptations to remote work, public policies and psychosocial consequences of the pandemic. In this first group, the boundaries were sometimes blurred, as certain intelligent data analysis applications can be understood as being on the borderline between one category and another.

The remaining three clusters (violet, green, yellow) represent different aspects related to biochemistry, vaccine and drug development, knowledge of the disease, treatments, and other general issues focused at the individual level with regard to the treatment of patients who are suffering or have suffered from COVID-19.

#### 3.2.1. Technology Applied to Adaptations of Different Sectors of Activity of Society to the Pandemic (Red Cluster)

The pandemic has required companies and services to adapt to remote working. Many companies have used different technological solutions related to the use of the Internet and communication, to cope with situations of isolation, quarantine and lockdown. After the lockdowns that were imposed in March 2020, many companies had to quickly and unexpectedly adapt their operations to teleworking, sometimes with large economic impacts (loss of revenue and unplanned investments) [55]. Artificial intelligence solutions have been developed to optimize efficacy, productivity and assess the digital fatigue of employees working remotely [56].

The pandemic has also forced many companies to readjust their customer service, online sales and telemarketing. Many sectors were already fully adapted but others, such as online food shopping [57], have had to adapt. Even sectors such as gambling have experienced a boom in this respect [58], and in a general view, all the marketing strategy has been redefined [59]. At this point, human work that cannot be replaced by an algorithm (known as ‘turking’) has become even more important, highlighting the limitations that companies may have [60].

This impact was also evident throughout the educational system, forcing teachers at all levels to abruptly move to online classes and to use all the technological power at their disposal. AI-assisted education is a promising field and is also known as educational intelligence. It is defined as “using data at several stages of the student’s life cycle to make informed decisions that have a positive impact on learning outcomes” [61]. AI technology can be used to develop and simulate human thinking and decision-making in a learning model. AI is used in adaptive education systems in the field of e-learning, including massive open online courses (MOOCs), educational data mining, and student analysis.

However, if there is one area in which adaptation to the pandemic situation has been a major challenge, it has been in the field of non-COVID-19 healthcare. The scarcity of public resources has led to a need to prioritize them. Electronic health records (EHRs) contain data that can be used to identify individuals’ clinical risk factors [62]. To meet the challenge of remote medical care (i.e., without the physical presence of the patient), Internet of Medical Things wearables can enable the delivery of AI-driven smart healthcare, essential services, and individualized clinical care [63].

#### 3.2.2. Artificial Intelligence Applied to Large-Scale COVID-19 Management Public Policies (Dark Blue Cluster)

A detailed view of this cluster shows that the common topics are the application of intelligent data analysis and related fields to outbreak prediction, modeling the spread of the disease, or screening for the virus on a large scale. This can be referred to collectively as epidemiology. Modeling and predicting the spread of COVID-19 using AI and machine learning (ML) techniques can provide valuable inputs for governments, health organizations, businesses, and individuals in terms of the management of the pandemic. Neural networks (NNs) have also played an important role in this respect. Both multi-layer feedforward NNs [64] and convolutional neural networks (CNNs) [65] have been used to predict cases. Other well-known algorithms have been tested, such as ARIMA (auto-regressive integrated moving average model) [66] and support vector machine (SVM), which are mainly used for the forecasting of time series data [67,68]. Several of these models have been applied as predictors of daily infections under different types of lockdown, thus helping in government decision making [69,70]. ML techniques have been successfully used to plan public policies [71].

Once a person has been diagnosed and confirmed with COVID-19, the next vital step is contact tracing to stop the spread of the disease. With this in mind, many infected countries are using mobile applications to carry out a digital contact tracing process, using a variety of technologies such as Bluetooth, global positioning system (GPS), contact data and smartphone tracking. A combination of big data and geographic information systems (GIS) is useful in this regard [72], as are IoT solutions [73] that follow the principles of other IoT-based disease management platforms [74]. Other technological solutions for social monitoring can also be implemented, such as the use of thermal cameras to detect individuals with fever [75].

#### 3.2.3. Data Analysis Applied to Psychosocial Issues and COVID-19 Pandemic (Light Blue Cluster)

The words that were identified in this cluster, such as ‘anxiety’, ‘negative sentiment’, ‘behavior’, ‘psychological distress’, relate to psychological issues arising from the unusual situation of the pandemic. It should not be forgotten that the pandemic, the resulting economic instability and in many cases lockdowns and quarantine situations have led to psychosocial problems that can be detected on a large scale by text mining from social media [76]. Loneliness has been one of the major consequences for people living alone and the elderly, and this can be mitigated using Information and Communications Technologies (ICT) interventions [77]. The impact of loneliness on social networks has also been studied using text mining strategies [78]. In addition, AI has been able to predict mental disorders in health care workers during the worst of the pandemic [79].

However, the enormous number of sources of information has led to a so-called “infodemic”, that is, “an over-abundance of information (some accurate and some not) that makes it hard for people to find trustworthy sources” [80]. Fortunately, ML and AI tools have been developed to prevent the propagation of false information over social networks and other media [81]. This “infodemic” can also be seen in the scientific field, as due to the enormous number of studies that have been published over the last year and a half, it is sometimes difficult to extract correct information [82].

#### 3.2.4. Drug Repurposing and Vaccines (Green Cluster)

A significant application of computer-supported medication repurposing is the treatment of new diseases such as COVID-19 by identifying drugs that were created to treat other diseases. Medication can be repurposed by leading methodical interaction between drug investigations and examining drug-target interactions, which can be achieved using AI-based tools [83]. AI has been perceived to affect drug advancement. As shown in a new report, the use of AI and big data can improve medical service frameworks and may have positive results in the drug market, and it has been anticipated by industry specialists that the creation of drugs through AI strategies will yield enhanced feedback [84]. A few organizations are presently using AI techniques to discover novel uses for late-stage drug competitors or to repurpose existing medications [85].

Natural language processing (NLP) is another aspect of AI that can be applied to the improvement of COVID-19 drugs. This strategy can be valuable in terms of extracting knowledge from text through the use of AI tools and looking for biomedical content related to drug repurposing [86].

AI and intelligent data analysis have also been crucial in vaccine development. ML techniques and AI are very effective for repetitive tasks that require large-scale information analysis, making them suitable for use in drug development. Deep learning has been a particularly important tool for predicting the properties and applications of medicinal chemicals that could inspire a response to an infection in the body [87]. Mechanizing this interaction would be immensely valuable, as this investigative process commonly requires long periods of experimentation and a large budget [88]. Scientists have been able to train models to predict which immunogenic sites to include in a vaccine, to allow the immune system to learn and become ready to encounter these particular antigens [89]. AI can also be helpful in recognizing antigens that have previously been identified in pathogens that may be similar to antigens for a new infection, thus further speeding up the process.

AI is playing a significant role in developing vaccines by facilitating an understanding of viral protein constructions and helping clinical specialists to scour several thousands of relevant research results at higher speeds than would otherwise be possible [90]. Knowledge about the structure of a virus can be instrumental in the development of a successful vaccine.

In their work on COVID-19, analysts at MIT’s Computer Science and Artificial Intelligence Laboratory (CSAIL) have focused on the spike proteins of the virus, a particular part of the virus which can act as a target. Their approach has suggested strategies for planning new peptide vaccines, assessing existing antibodies, and expanding existing immunization plans [91].

Spike proteins are important in current COVID-19 immunization treatment, as they incorporate antigens that can be identified and attacked by the immune system [92].

#### 3.2.5. Diagnosis and AI-Aided Tests (Yellow Cluster)

In this cluster, words such as ‘detection’, ‘image’, ‘chest X-ray image’, ‘diagnosis’ and ‘pneumonia’ suggest a general topic related to the diagnosis of the disease and the associated medical tests. The cluster contains works on the application of deep learning and ML classifiers to these issues.

Chest computed tomography (CT) images can help in the diagnosis of COVID-19. The characteristic features shown by COVID-19 patients, such as vascular thickening, fine reticular opacity, ground-glass opacity and peripheral distribution, can be detected by an AI-based classifier [93]. A CNN has been used for this purpose, with the aim of helping medical staff to make more accurate decisions [94].

Other solutions involving smart devices based on IoT and ML techniques have also been used to help in the diagnosis of COVID-19 [95].

These tools have been applied to COVID-19 to identify the illness and the relevant risk factors, based on features such as clinical presentation, laboratory results, age, weight and comorbidities, using SVM [96], and then estimating the risk of mortality [97].

#### 3.2.6. Disease Progression (Violet Cluster)

This cluster is related to the evolution of the disease in a given patient and is focused on the individual level. Concepts such as ‘admission’, ‘comorbidity’, and ‘predictor’ are associated with this idea, as are other terms related to treatment (excluding drug treatment), such as ‘mechanical ventilation’ or ‘intensive care unit’, all of which revolve around the patient. AI has played an important role in the critical care of COVID-19 patients. Several studies [98] have shown that NNs, ML and DL can help in intensive care unit (ICU) decision making regarding treatment, risk stratification and management, and the deterioration of patients in the emergency department [99]. AI can also improve assessments of the severity of pneumonia and predict the need for mechanical ventilation [100].

#### 3.2.7. Equivalences with WoS Source

**Figure 8:**
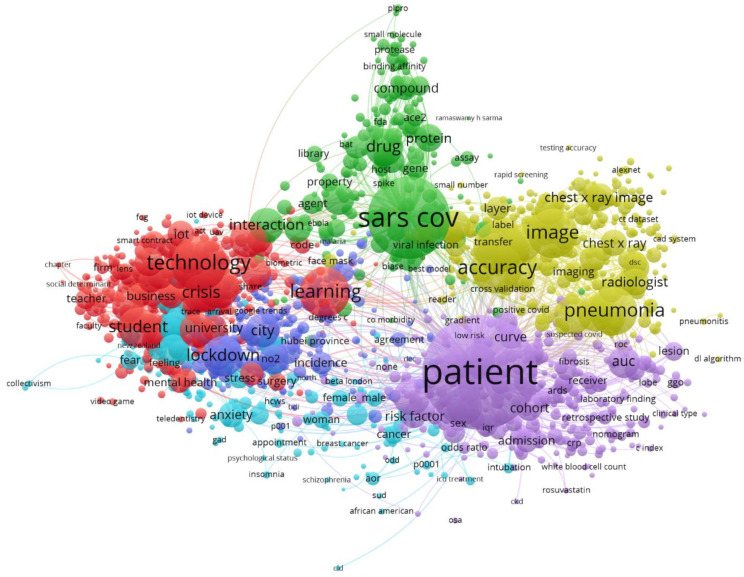
Topic mapping and clustering based on affinities (source WoS).

In the same way, similar topics can be seen in the WoS data. The six clusters described above have been drawn in Figure 8, using the same colors for better identification. The fact that both databases (Scopus and WoS) present the differences described in Section 2.4.1. means that the keywords automatically identified are not the same, although clear equivalences can be seen. For example, in the red cluster one can clearly identify the concepts “learning” or “student”, and in similar areas “anxiety” or “mental disorder” (light blue). The cluster in dark blue is not so clearly identified.

Other clusters follow with a clear equivalence, such as yellow, green and violet, clearly oriented, (respectively), to diagnosis (“image”, “detection”), biochemical development (“drug”, “protein”, “vaccine”), or in the case of violet cluster we see concepts equally related in both cases to the evolution of the patient.

### 3.3. Topics Variation along Time

**Figure 9:**
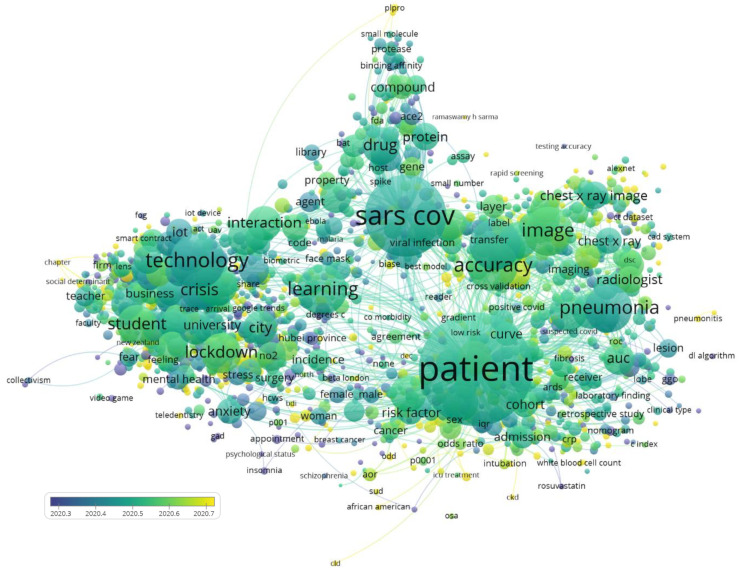
Topic mapping and visualization overlaid with the average year of publication (source WoS).

Once an analysis of topics had been carried out and their affinities and clusters had been obtained, an overlay visualization was used to superimpose the average publication date for each topic. This strategy made it possible to see which lines of research were first developed with regard to the application of AI to the management of COVID-19, and which topics are currently the focus of research interest. Figure 9 illustrates this issue, where the darker circles (blue, dark green) relate to the early part of 2020, and the light green and yellow circles represent more recent work.

Although this technique was developed to make it possible to observe evolutions over decades of research, in our specific case the analysis is necessarily limited to publications from 2020 and the first part of 2021. Nevertheless, an evolution can be seen. Applications related to public policy and curbing the spread of the virus were the first to be developed [101] (corresponding to the red, dark blue and light blue clusters in Figure 7 and Figure 8). This is as expected, as these were direct applications of strategies that were already known and had been applied to other fields (social tracing, teleworking, tele-education, etc.), and public policy management. Similarly, sentiment analysis using social networks was a field that had previously been developed; some research was carried out immediately after the first lockdowns [102], and other strategies were applied with the aim of managing lockdowns and quarantines [103], which was a priority in March 2020.

We also found scientific publications related to pharmacological development in which a great deal of research on repurposing had been carried out [104]. The development of assisted vaccines and support for these via AI could also be observed in this intermediate zone (light blue), while innovations in disease diagnosis and progression appeared in more recently published work.

The above ideas represent a general interpretation, and isolated points could be seen in all thematic clusters that corresponded to topics that were developed at different times to those in their vicinity.

It should be borne in mind that the colors assigned to the map in Figure 9 represent the average dates of publication for manuscripts related to each topic; this does not mean that there were no publications on a given topic that were pioneering or very recent.

### 3.4. Citations and Highly Cited Elements

The most frequently cited journals and the most highly cited papers and authors can be used to identify the most important elements of the research in the field of COVID-19 and disruptive technologies.

#### 3.4.1. Citation by Source

In order to analyze the citations by source, the 10 journals with the most citations were extracted from Scopus (Table 2). We can distinguish several types of journals at a glance, corresponding to certain categories: journals of a multidisciplinary nature and journals devoted exclusively to medicine (medical imaging). The vast majority of these specialized in medical informatics, biomedical, or bioinformatics, and the most frequently cited journals in AI applications to COVID-19 management were therefore specialized publications.

We can see that the journal with the most citations was *Lancet*, with 9047 citations from 26 published papers. The second most highly cited was *Radiology,* with 2722 citations in 20 documents and the third was the *International Journal of Environmental Research and Public Health*, with 2310 citations and 426 papers. Some journals had a large number of citations from numerous publications on this issue, while others were cited in large numbers only by a few papers. The citation/document ratio was therefore calculated to evaluate this aspect. It should be noted that *Lancet* had an exceptional ratio of 347.96 citations per paper.

Of the 10 journals selected, three were open access, a type of publication that facilitates access and consultation, but many others support in some way also this type of publication.

#### 3.4.2. Citation by Number of Papers

When studying the papers with the most citations, it became obvious that since the beginning of the pandemic, research published earlier had had a greater impact. The 10 most cited papers (Table 3) were all from 2020, and the majority of these were published in March and April of that year.

Predictive models for the diagnosis and prognosis of patients and the application of AI to X-ray image recognition or CT images topped this list. Others dealt with the social management of the pandemic using intelligent data analysis. It is notable that the fourth most frequently cited paper dealt with the psychosocial consequences of the pandemic through the analysis of social network data.

### 3.5. Co-Citation Analysis

Co-citation analysis is a statistical method that can identify idle connections among authors and/or journals and express these visually in the form of co-citation clusters, to facilitate an understanding of this information. This approach is based on the idea that papers in journals or by researchers who are often co-cited are likely to address similar or related ideas. Co-citation occurs when two different published manuscripts receive a citation from a third document.

#### 3.5.1. Co-Citation by Source

**Figure 10:**
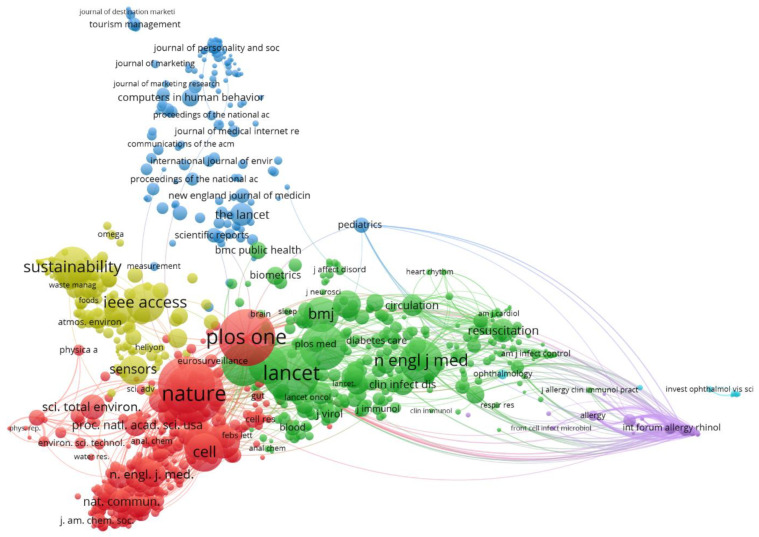
Co-citation mapping by sources (source Scopus).

If we examine the co-citations by source (journal), we can see that these are organized into five clusters (Figure 10), which can be described as follows.

The yellow cluster represents generalist and multidisciplinary journals, such as *Sustainability*. These are typically publications on topics related to AI applications to COVID-19 management in the social domain, although other topics can also be found in this cluster.

The green cluster represents journals containing articles dealing with ML applications and intelligent analysis focused on diagnosis. This includes *Lancet* or *Radiology*.

The red cluster represents specialized medical journals such as *Nature* or *Cell*. The issues in this cluster are related to the use of deep learning in the course of the vaccine and redefining other drugs.

Finally, the blue cluster represents other papers connecting COVID-19, emerging technologies and other issues, such as tourism, sociology, psychology or marketing. The violet cluster in Figure 10 group journals dedicated to allergy, as certain symptoms related to the course of the disease may be similar (asthma, rhinitis, skin rashes) [115].

**Figure 11:**
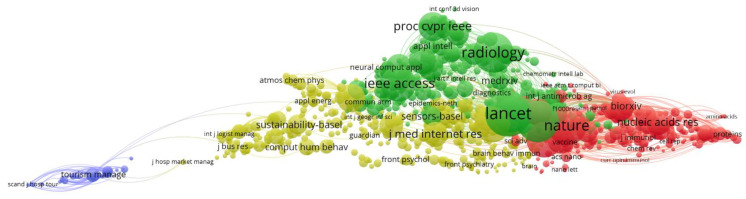
Co-citation mapping by sources (source WoS).

Using WoS data the results are somewhat similar (Figure 11), with the absence of the violet cluster. The blue cluster is much more concentrated, possibly due to the greater presence of this type of sociology journals in Scopus.

#### 3.5.2. Co-Citations by Author

**Figure 12:**
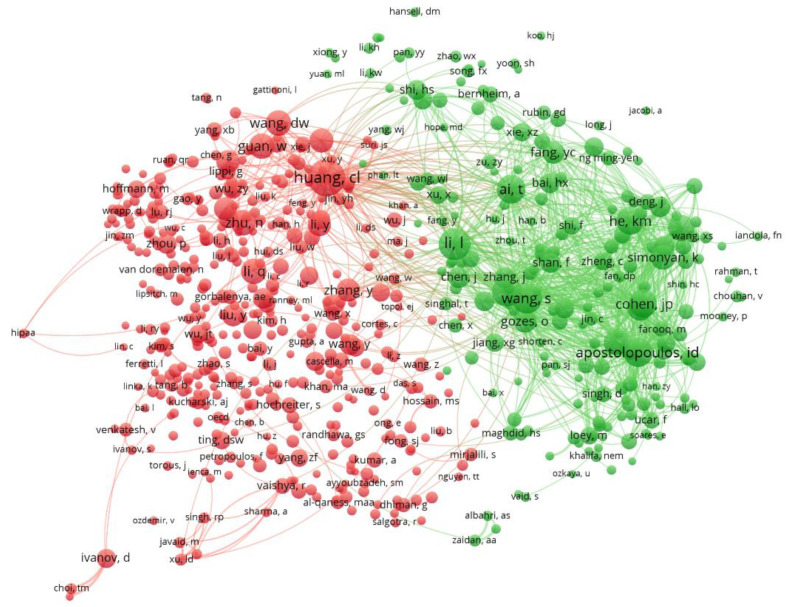
Co-citation mapping by authors (source WoS).

An analysis of co-citations can help in understanding the connections between authors. Authors that are connected by co-citations are presumed to have some kind of relationship, such as their discipline, subject, country or affiliation. Clusters of co-citations by authors formed two distinct groups; in this case, these were related to the subjects of research, so this is the way that in our case co-citations are structured (Figure 12). The first (shown in red) consisted of authors in the field of biochemistry, genetics or pharmacology who had published research on the applications of intelligent data analysis within these areas. The list of authors was headed by Chaolin Huang (Wuhan Institute of Virology, Chinese Academy of Sciences, Wuhan, China). This author had a total of 405 co-citations and was the lead author of one of the basic reference papers on COVID-19 describing the clinical features of patients [116]. This feature set has formed the basis of numerous ML algorithms for diagnosis and has been cited almost 30,000 times according to Google Scholar [117].

The second cluster was made up of authors who had focused on applications of AI to diagnosis, mainly by applying deep learning to the recognition of lung images to check the course of the disease. In this area, we find Ioannis D. Apostolopoulos (University of Patras, Department of Medical Physics, Greece), who applied a CNN to X-ray images to perform automatic diagnosis. The paper in which he explained this research was highly cited [118], and this author had a total of 251 co-citations.

Other co-cited authors in this second cluster with high numbers of co-citations included Shuai Wang (Department of Molecular Radiation Oncology, Tianjin Medical University, China) who applied deep learning to CT images to screen for COVID-19 in lungs [119], and Lin Li, (Department of Radiology, Wuhan Huangpi People’s Hospital, Wuhan, China) with a highly cited paper in which AI was applied to a large database of chest CT images to identify COVID-19 [120].

Regarding this second cluster, if we take into account that some topics are more frequently co-cited than others, we can conclude that the application of powerful AI tools to diagnosis dominates this group.

## 4. Conclusions

Since the outbreak of the COVID-19 pandemic, great efforts have been made to minimize its effects. The search for effective treatments, vaccines, and social management mechanisms has intensified around the world. AI and other emerging technologies have undoubtedly played an important role, offering new perspectives and strategies to researchers.

This has meant that scientific production has undergone explosive growth in terms of new data, approaches and results, which is difficult to manage. As discussed in this paper, the publication of papers related to COVID-19 and AI technologies continues to increase; the total output so far in 2021 already represents double the entire production in 2020. As highlighted in this work, the countries with the greatest output in this respect are the USA, China and India. It is notable that almost all of the top 10 countries with the greatest volume of scientific production on the topic of COVID-19/AI are countries with high levels of resources; this demonstrates the need for funding and economic support for research. On the other hand, we have shown that there has been a high level of collaboration between countries, in an increasingly globalized world. In addition to collaborations between the USA and China, there was a great deal of cooperation between neighboring and geographically close countries. This indicates that the ease of physical movement and cultural affinities may lead to greater collaboration between countries on the same continent. The COVID-19 pandemic has increased the use of teleworking, and it remains to be seen whether remote collaboration will change this assessment in the coming years.

These social variations have been a point of study identified in this work, as a relevant topic. Others related to public policies and ICT applications to management have also been studied. In addition to these three clusters, three other clusters have been highlighted as important, and these are related to health and sanitary aspects. Biochemistry, pharmacology and vaccine development using intelligent data analysis techniques were topics of great importance, as were other diagnostic techniques using deep learning and approaches to patient and disease management. It can be seen how, in a temporal evolution, the latter are currently more developed. However, the topics identified here were sometimes difficult to separate, and there were “borderline” topics that could have been included in more than one cluster.

All the publications identified here have been published in different journals, usually with a high level of impact. Some journals achieved a large number of citations, either due to the overall quality of their articles or because a single paper was frequently cited. It should also be pointed out that although many papers on the subject of AI applications to COVID-19 were published in journals that were specific to medicine or computer science, some were published in multi-disciplinary journals. A large number of papers were highly cited thanks to the open access model, which allows for free access to information.

Our co-citation analysis showed that, in general, papers in journals that focused on the same subject were commonly co-cited. We were therefore able to clearly show how these were organized by topic, from AI applications to COVID-19 management, diagnostics and medicine/biochemistry. An analysis of co-citations by author led to the same conclusion: authors of papers in the fields of biochemistry and pharmacology tended to cite each other, and authors of papers on more direct applications of intelligent data analysis also tended to cite each other.

The use of two data sources, Scopus and WoS, has helped us to compare the conclusions obtained. It has been observed that the samples have been of very different sizes, and even so, equivalent groupings have been observed. This indicates that the WoS sample, although smaller, is still representative of research in emerging technologies applied to COVID-19. However, in some cases there may have been discrepancies, due to the different coverages of each database.

This paper has presented an outline of the research that has been carried out over a period of approximately a year and a half, in relation to the application of intelligent data analysis to issues arising from the COVID-19 pandemic. In future work, we intend to continue our analysis of this interesting topic via bibliographic analysis, in order to develop a better understanding of the collaborations between researchers, the evolution of scientific production, and the trends in research in this area.

## Figures and Tables

**Table 1:** Search criteria.

Searching Criteria		
		machine learning OR
		artificial intelligence OR
		deep learning OR
		neural network OR
		big data OR
		internet of things OR
		cloud computing OR
		edge computing OR
		quantum computing OR
		virtual reality OR
Covid * OR		augmented reality OR
sars-cov-2 OR		cyber security OR
2019-ncov OR	AND	biometrics OR
Severe acute		5G OR
respiratory syndrome)		natural language processing OR
		feature selection OR
		random forest OR
		support vector machines OR
		decision trees OR
		blockchain OR
		cloud computing OR
		genetic algorithm OR
		gradient boosting OR
		k nearest neighbors OR
		naïve bayes

Note: The * has been used as a wildcard character.

**Table 2:** Citation by source.

Citations	Journal	Documents	Ratio (cit/doc)	Categories
9047	Lancet	26	347.96	Medicine, General and Internal
2722	Radiology	20	136.10	Radiology, Nuclear Medicine and Medical Imaging
2310	International Journal of Environmental Research and Public Health (*)	426	5.42	Public, Environmental and Occupational Health-Environmental Sciences
2156	Nature	30	71.87	Multidisciplinary
2142	Chaos Solitons and Fractals	120	17.85	Physics, Multidisciplinary-Mathematics, Interdisciplinary Applications-Physics, Mathematical
2103	Science of the Total Environment	70	30.04	Environmental Sciences
1695	Journal of Medical Internet Research (*)	294	5.77	Health Care Sciences and Services-Medical Informatics
1660	Cell	21	79.05	Cell Biology, Biochemistry and Molecular Biology
1621	Clinical Infectious Diseases	9	180.11	Microbiology, Infectious Diseases and Immunology
1517	Plos One (*)	355	4.27	Biology, Multidisciplinary Sciences

(*) Open access journals.

**Table 3:** Citation by number of papers.

Document Title	Authors	Year	Source	Cited by
Genomic characterization and epidemiology of 2019 novel coronavirus: implications for virus origins and receptor binding [105]	Lu, R., Zhao, X., Li, J., (...), Shi, W., Tan, W.	2020	The Lancet, 395 (10,224), pp. 565–574	4444
Correlation of Chest CT and RT-PCR Testing for Coronavirus Disease 2019 (COVID-19) in China: A Report of 1014 Cases [106]	Ai, T., Yang, Z., Hou, H., (...), Sun, Z., Xia, L.	2020	Radiology, 296 (2), pp. E32–E40	2074
In vitro antiviral activity and projection of optimized dosing design of hydroxychloroquine for the treatment of severe acute respiratory syndrome coronavirus 2 (SARS-CoV-2) [107]	Yao, X., Ye, F., Zhang, M., (...), Tan, W., Liu, D.	2020	Clinical Infectious Diseases, 71 (15), pp. 732–739	1292
Remdesivir in adults with severe COVID-19: a randomized, double-blind, placebo-controlled, multicenter trial [108]	Wang, Y., Zhang, D., Du, G., (...), Cao, B., Wang, C.	2020	The Lancet, 395 (10,236), pp. 1569–1578	1290
How will country-based mitigation measures influence the course of the COVID-19 epidemic? [109]	Anderson, R.M., Heesterbeek, H., Klinkenberg, D., Hollingsworth, T.D.	2020	The Lancet, 395 (10,228), pp. 931–934	1159
A SARS-CoV-2 protein interaction map reveals targets for drug repurposing [110]	Gordon, D.E., Jang, G.M., Bouhaddou, M., (...), Shoichet, B.K., Krogan, N.J.	2020	Nature, 583 (7816), pp. 459–468	1068
SARS-CoV-2 Receptor ACE2 Is an Interferon-Stimulated Gene in Human Airway Epithelial Cells and Is Detected in Specific Cell Subsets across Tissues [111]	Ziegler, C.G.K., Allon, S.J., Nyquist, S.K., (...), Xu, Y., Zhang, K.	2020	Cell, 181 (5), pp. 1016–1035.e19	741
Quantifying SARS-CoV-2 transmission suggests epidemic control with digital contact tracing [112]	Ferretti, L., Wymant, C., Kendall, M., (...), Bonsall, D., Fraser, C.	2020	Science, 368 (6491)	706
Prediction models for diagnosis and prognosis of covid-19: Systematic review and critical appraisal [113]	Wynants, L., Van Calster, B., Collins, G.S., (...), Moons, K.G.M., Van Smeden, M.	2020	The BMJ, 369, m1328	651
Online mental health services in China during the COVID-19 outbreak [114]	Liu, S., Yang, L., Zhang, C., (...), Hu, S., Zhang, B.	2020	The Lancet Psychiatry, 7 (4), pp. e17–e18	623

## Data Availability

All the data is available at Web of Science. https://www.webofknowledge.com and Scopus https://www.scopus.com (accessed on 1 August 2021).
